# Supplementary material for: Pharmacological stimulation of infralimbic cortex after fear conditioning facilitates subsequent fear extinction
Source: Neuropsychopharmacology. 2024 Aug 13;49(13):1951–7. doi: 10.1038/s41386-024-01961-9 (PMC11480363; doi:10.1038/s41386-024-01961-9)
Supplement: Supplementary file 1 — Supplementary material [file 41386_2024_1961_MOESM1_ESM.pdf]

Supplemental Material

Picrotoxin spares conditioned freezing during the first extinction trial

In four experiments we found that picrotoxin infused from 1 hour to 13 days after fear conditioning produced dramatic impairments in conditioned freezing during subsequent extinction sessions. Freezing during the first 5-trial block was consistently reduced in each experiment. However, it is important to know whether freezing was lower in response to the first trial of each extinction session, because this trial provides an index of fear memory retrieval independent of extinction. To this end, we examined freezing during each of the first five conditioning trials for all the experiments. Each trial consisted of a 10-sec tone and a 30-sec inter-trial interval (ITI). We only compared animals that received vehicle or PIC in the IL (BDNF- and ANISO-treated animals were excluded from this analysis).

As shown in Figure S1 (A), conditioned freezing was similar in VEH- and PIC-treated rats during the first CS presentation of the extinction session but diverged during the first ITI in each of the four experiments. Figure S1 (B) shows freezing during the first CS in each experiment. In none of the experiments did the difference between VEH and PIC groups reach significance (p's > 0.052). These analyses suggest that even though the PIC manipulations generally reduced freezing during the first 5-trial extinction block, the infusions spared freezing to the first CS of the extinction session. Picrotoxin infusions into the IL appears to accelerate extinction of freezing to the CS rather than producing a general reduction in the expression of the conditioned freezing response or retrieval of the fear memory.

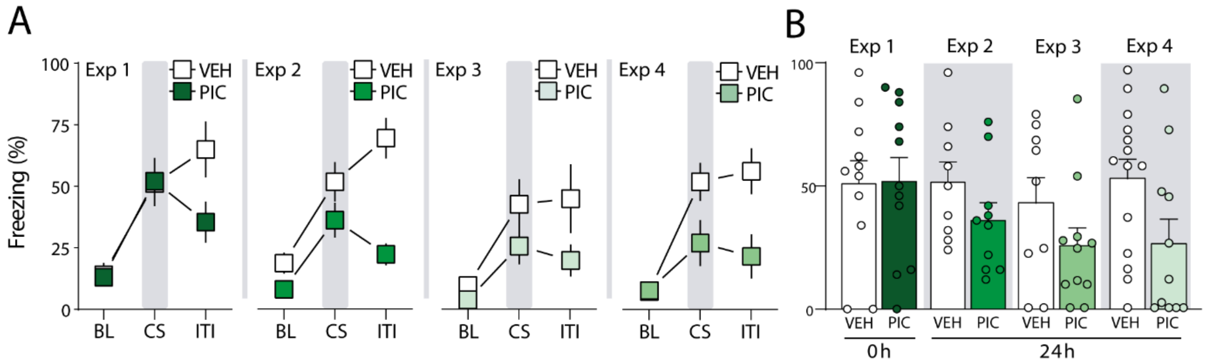

Figure S1. Picrotoxin does not disrupt fear recall on the first extinction trial. (A) Freezing data during the first extinction trial during the baseline (BL) period, the conditioned stimulus (CS), and post-CS intertrial intertrial (ITI) for each experiment. Freezing during the CS is highlighted by gray bars. **(B)** Average CS freezing in each of the four experiments in VEH- and PIC-treated rats. There were no group differences in any of the experiments (Exp 1: $p = 0.94$; Exp 2: $p = 0.15$; Exp 3: $p = 0.19$; Exp 4: $p = 0.052$). Exp = experiment; PIC = picrotoxin; VEH = vehicle.
